# Supplementary material for: MCAM contributes to the establishment of cell autonomous polarity in myogenic and chondrogenic differentiation
Source: Biol Open. 2017 Sep 18;6(11):1592–601. doi: 10.1242/bio.027771 (PMC5703611; doi:10.1242/bio.027771)
Supplement: Supplementary information [file biolopen-6-027771-s1.pdf]

A

```
10T1/2  TACCCACGCCCG--ACCTGGTGGAGGCAGAAGTGGGCAGCACAGCCCT
C149    TACCCACGCCCG-AACCTGGTGGAGGCAGAAGTGGGCAGCACAGCCCT
C149    TACCCACGCCCGCGACCTGGTGGAGGCAGAAGTGGGCAGCACAGCCCT
```

Chr9: 44,136,557-44,136,558insA

Chr9: 44,136,557-44,136,558insCG

```
10T1/2  TACCCACGCCCG-ACCTGGTGGAGGCAGAAGTGGGCAGCACAGCCCT
C164    TACCCACG-----ACCTGGTGGAGGCAGAAGTGGGCAGCACAGCCCT
C164    TACCCACGCCCGTACCTGGTGGAGGCAGAAGTGGGCAGCACAGCCCT
```

Chr9: 44,136,554-44,136,557delCCCG

Chr9: 44,136,557-44,136,558insT

```
10T1/2  CTCTCCTTCAGGGCAGCAACGGTGACAAGAGGGCTCCAGGAGACCAGGTAGGAGGCAGTTCCTGAGGCCAGGGC
U125    CTCTCCTTCAGGGCAGCAACGGTGACAA-AGGGCTCCAGGAGACCAGGTAGGAGGCAGTTCCTGAGGCCAGGGC
U125    CTCTCCTTCAGGGCAGCAA-------------------GGAGACCAGGTAGGAGGCAGTTCCTGAGGCCAGGGC
U125    CTCTCCT--------------------------------------------------------GAGGCCAGGGC
```

Chr9:44,141,374delG

Chr.9:44141365-44141383delCGGTGACAAGAGGGCTCCA

Chr9:44,141,353-44,141,408delTCAGGGCAGCAACGGTGACAAGAGGGCTCCAGGAGACCAGGTAGGAGGCAGTTCCT

B

```
10T1/2: MGLPKLVCVFLFAACCCCRRAAGVPGEEKQPVPTPDLVEAEVGSTALLKCGPSRASGNFSQVDWFLIHKERQILIFRVHQGKGQR...
C149:   MGLPKLVCVFLFAACCCCRRAAGVPGEEKQPVPTPEPGGGRSGQHSPSQVWPLTGLRQLQPSGLVFDSQGEADTDFPCAPRQGPAGTW-STOP
C149:   MGLPKLVCVFLFAACCCCRRAAGVPGEEKQPVPTPATWWRQKWAAQPFSSVAPHGPQATSAKWTGF-STOP
C164:   MGLPKLVCVFLFAACCCCRRAAGVPGEEKQPVPTTWWRQKWAAQPFSSVAPHGPQATSAKWTGF-STOP
C164:   MGLPKLVCVFLFAACCCCRRAAGVPGEEKQPVPTPVPGGGRSGQHSPSQVWPLTGLRQLQPSGLVFDSQGEADTDFPCAPRQGPAGTW-STOP

10T1/2: ...EEMALLQGSNGDKRAPGDQGEKYIDLRH-STOP
U125:   ...EEMALLQGSKETRERNTSI-STOP
U125:   ...EEMALLQGSNGDKGLQETRERNTSI-STOP
U125:   ...EEMALLREIHRSEALDGSHCTARSSLLRLHPKLKPPEGQQGRATLNPPPAHQV-STOP
```

Fig. S1. Genome editing of *Mcam* locus. (A) CRISPR-Cas9 induced DNA sequence changes (in blue) and their genomic locations. (B) Amino acid substitutions (magenta) in MCAM introduced by CRIPSR-Cas9 genome editing. YIDL endocytosis motif highlighted in bold.

Biology Open • Supplementary information

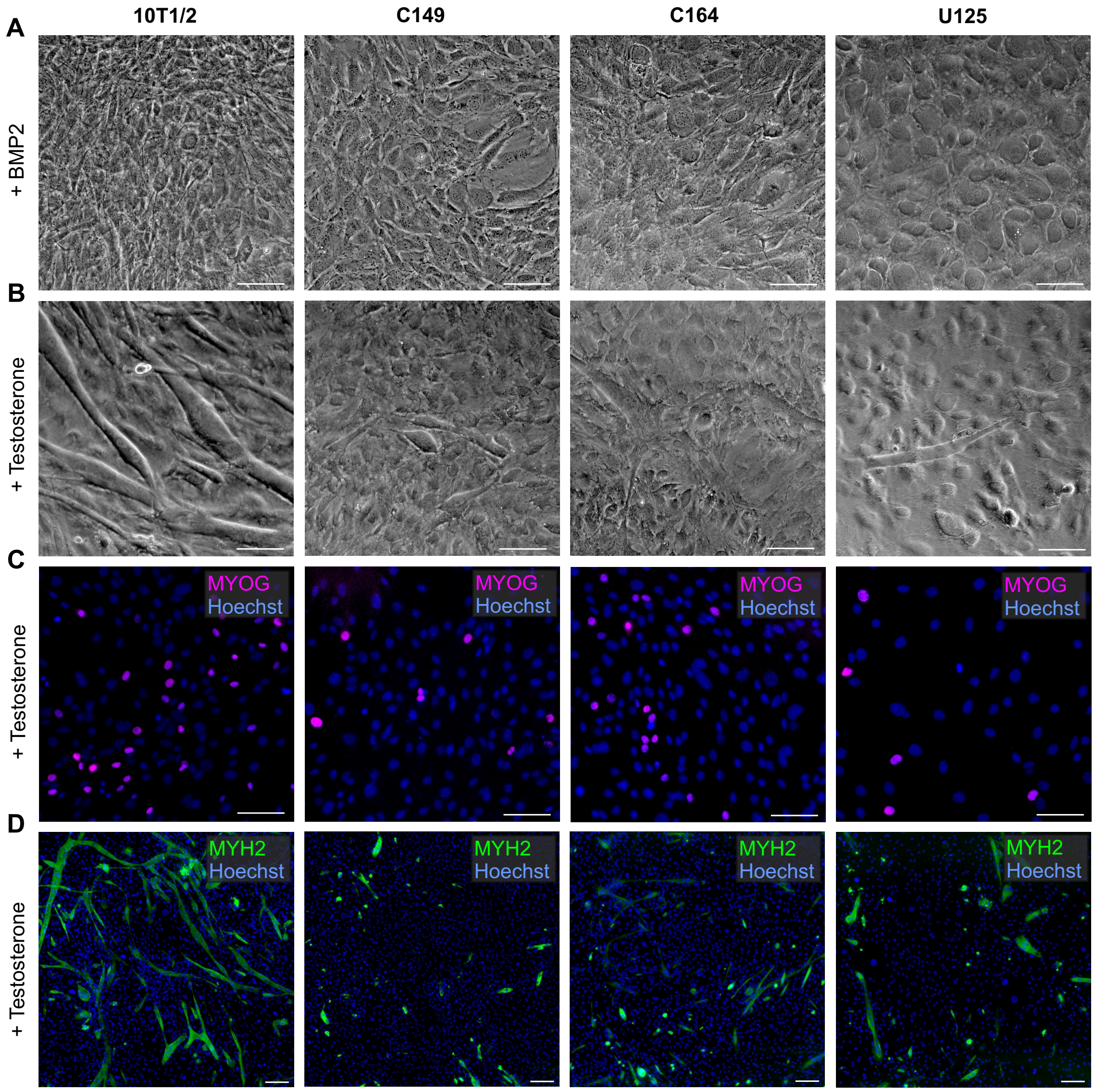

Fig. S2. Loss of MCAM function impairs differentiation. (A) Phase contrast images of wild type 10T1/2, MCAM knockout C149 and C164 cell lines and MCAM endocytosis motif mutant cell line U125 treated for 7 days with BMP2 to trigger osteochondrogenic differentiation. (B) Same cell lines exposed for 7 days to testosterone to induce myogenic differentiation. (C) After 4 days of testosterone exposure MYOG was upregulated in both wild type and MCAM loss of function cell lines. (D) Low magnification images show impaired formation of elongated myotubes in CRISPR-Cas9 edited cells exposed for 7 days to testosterone. Scale bars: A-B, 100 µm; C-D, 200 µm.

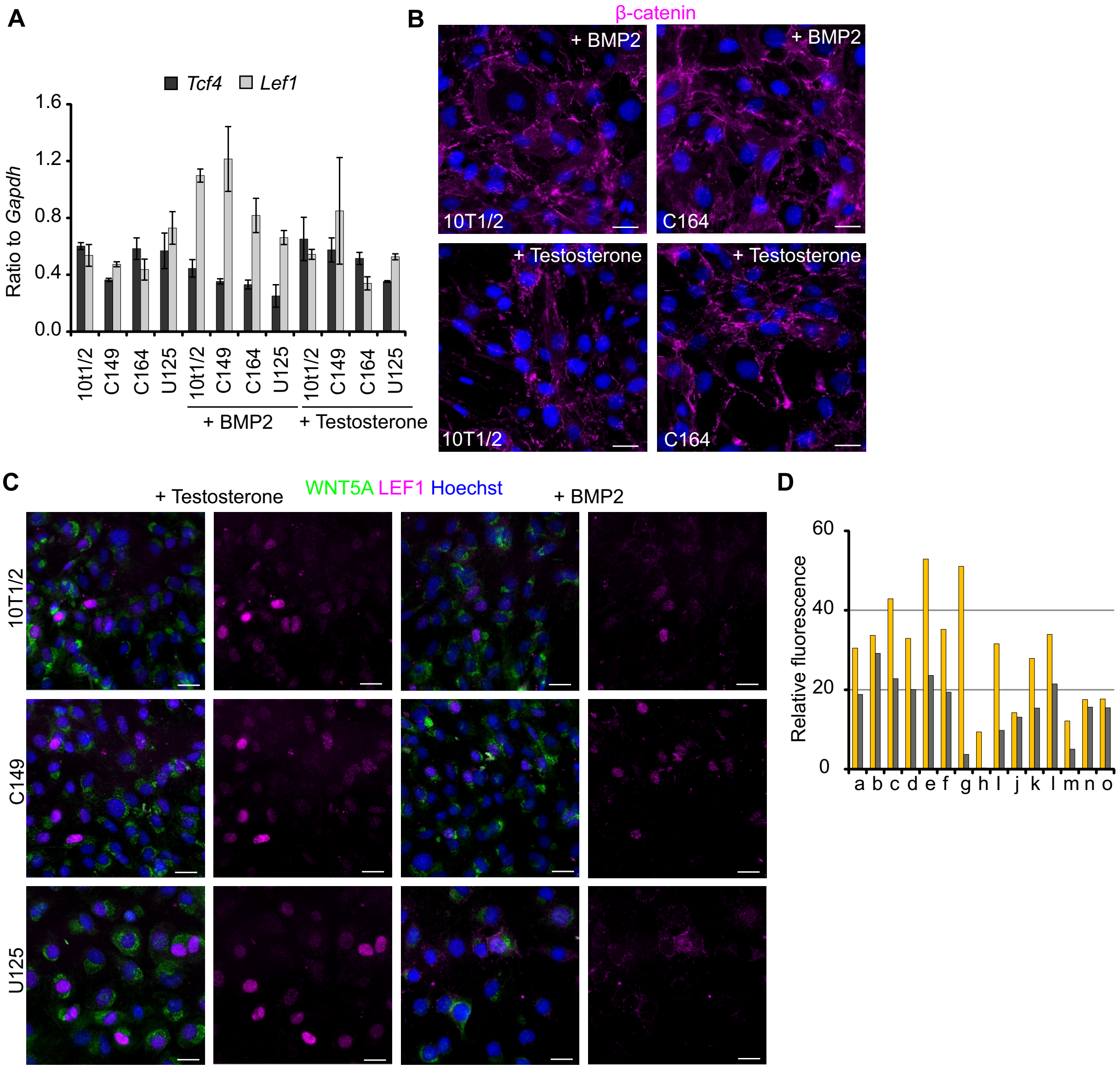

Fig. S3. MCAM knockout does not affect canonical WNT signalling. (A) *Tcf4* and *Lef1* were expressed in the 10T1/2 cells exposed for 7 days to BMP2 and testosterone, but their expression levels did not depend on *Mcam* (RT-qPCR analysis (mean +/- SEM)). Expression relative to housekeeping gene *Gapdh*. (B) MCAM knockout did not induce nuclear translocation of β-catenin. Cells were exposed for 4 days to BMP2 or testosterone. (C) Differentiating cells express simultaneously non canonical WNT5A and canonical WNT pathway mediator LEF1. (D) Quantification of MCAM staining intensity in 15 myotubes (a-o) in E17.5 mouse embryonic limbs. The intensity was calculated in equal areas on both ends of the cell, demonstrated in yellow and grey. There is on average 4.6 fold difference in staining intensity (SEM=1.5). Scale bars: 20 μm.

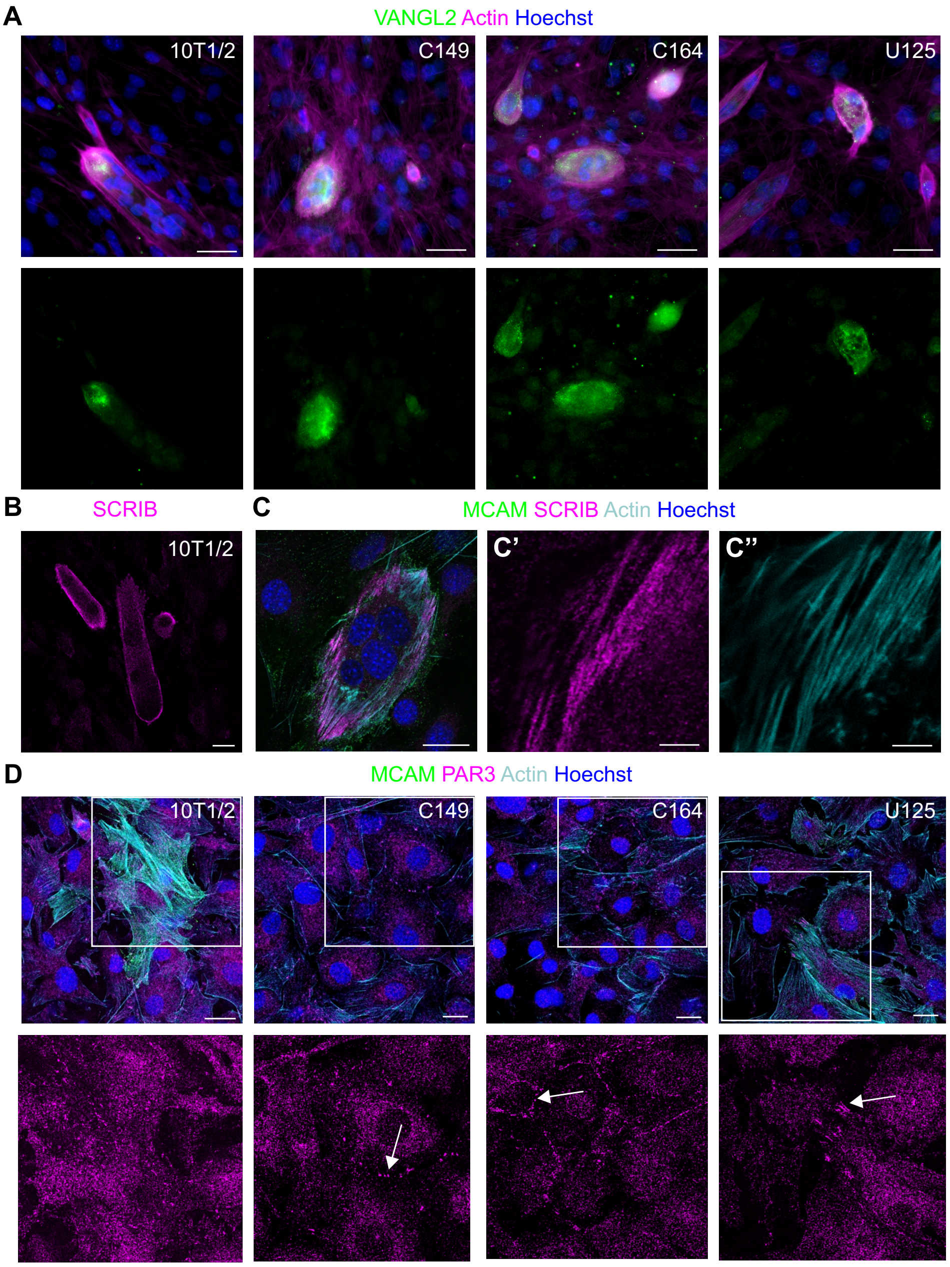

Fig. S4. MCAM regulates cell autonomous polarity. (A) Low magnification images showing asymmetric distribution of VANGL2 in wild type 10T1/2 cells but uniform localization in C149, C164 and U125 cells. Cells were exposed to testosterone for 7 days to induce myogenesis. Separate images for VANGL2 are shown below. (B) Asymmetric distribution of SCRIB in wildtype myotube. (C) In MCAM endocytosis motif mutant U125 cell line SCRIB colocalizes with cytoplasmic actin filaments instead of being targeted to the cell cortex. (C'-C") High magnification images of SCRIB and actin staining. (D) In 10T1/2 cells treated for 7 days with BMP2 PAR3 remained primarily cytoplasmic, whereas in MCAM mutant cell lines (C149, C164, U125) it showed frequent cortical targeting (arrows). Separate PAR3 channel of the boxed area is shown below. Scale bars: A, B, C, D, 25 µm; C'-C" 5 µm.

Biology Open • Supplementary information
